# Neural Correlates of Time Versus Money in Product Evaluation

**DOI:** 10.3389/fpsyg.2012.00372

**Published:** 2012-10-01

**Authors:** Sebastian Lehmann, Martin Reimann

**Affiliations:** ^1^Otto-von-Guericke-UniversityMagdeburg, Germany; ^2^University of Southern CaliforniaLos Angeles, CA, USA

**Keywords:** time-versus-money effect, priming, product evaluations, insula, functional magnetic resonance imaging, consumer neuroscience, decision neuroscience

## Abstract

The common saying “time is money” reflects the widespread belief in many people’s everyday life that time is valuable like money. Psychologically and neurophysiologically, however, these concepts seem to be quite different. This research replicates prior behavioral investigations by showing that merely mentioning “time” (compared to merely mentioning “money”) leads participants to evaluate a product more positively. Beyond this finding, the present functional magnetic resonance imaging (fMRI) experiment provides novel insight into the neurophysiological underpinnings of this behavioral effect by showing that more positive product evaluations in the time primes (compared to money primes) are preceded by increased activation in the insula. Our data, therefore, support the idea of a time mindset that is different from a money mindset. Studies on the functional neuroanatomy of the insula have implicated this brain area in distinct but related psychological phenomena such as urging, addiction, loss aversion, and love. These functions imply greater personal connection between the consumer and a target subject or object and, thus, help explain why time-primed consumers rate products more positively.

## Introduction

Franklin (1748/1961) once wrote: “Remember time is money,” suggesting that both concepts are economically equivalent (Becker, 1965; Okada and Hoch, 2004; DeVoe and Pfeffer, 2007; Zauberman et al., 2009). Yet, in psychological terms, time and money seem to be quite different. Several investigations have provided evidence that the concepts of “time” and “money” alter behavior in different ways. For example, Saini and Monga (2008) showed that decision-making is more heuristic in situations that require spending time than in situations that involve spending money. The authors argued that heuristics are used more for time because consumers’ time expenditures are harder to account for than those for money. Liu and Aaker (2008) explored the behavioral consequences of time compared to money in the context of charitable giving, showing that asking for time first (i.e., the “time-ask”), compared to asking for money first (i.e., the “money-ask”), increases the subsequent amount of money donated to the charity. The authors argued that time and money each may have activated a “mindset” that is different from the other: while thoughts of spending time for the charity may have activated a more emotional “mindset,” in which collective motives, goals of emotional well-being, and beliefs of personal happiness became salient, thoughts of giving money to the charity could have activated a value-maximization “mindset” that separated the donor from the charity psychologically and decreased beliefs of personal happiness (in this research, we use the term “mindset” following the work of Mogilner and Aaker, 2009). Building on the “time-ask effect,” Mogilner and Aaker (2009) revealed a “time-versus-money effect,” in which a time prime led to more favorable product attitudes. The authors contended that the activation of the concept of time (versus money) increased the focus on product experiences (versus product possessions) and, thus, augmented a personal connection between consumer and product. This connection, in turn, improved attitudes toward the product, increased the willingness-to-pay for the product, and influenced consumers’ decisions to actually buy the product.

While prior research has made progress in increasing the understanding of the differential behavioral effects of time versus money, the psychological and neurophysiological underpinnings of priming consumers with time compared to those of priming them with money are much less clear. For example, while Liu and Aaker (2008) argued that priming time leads to an emotional mindset while priming money triggers a value-maximization mindset, other investigators have provided some opposing evidence. In particular, Dunn et al. (2008) showed that spending money on others as compared to spending it on oneself promotes happiness, which indicates the involvement of an emotional mindset rather than a value-maximization one. As such, it is necessary to shed more light on the underlying neurophysiological mechanisms of time and money in order to understand *how* these concepts operate psychologically and differentially affect downstream behavior.

The present research utilizes functional magnetic resonance imaging (fMRI) to investigate the neurophysiological underpinnings of time primes versus money primes and their consequences for product evaluation. fMRI offers some methodological advantages over self-report measurement as it (1) permits interpretation of psychological processes in the brain as they are taking place,(2) enables measurement of non-conscious processes, and (3) allows for localization and differentiation of concepts that may seem subjectively similar but are actually processed differently (Shiv, 2007; Reimann et al., 2011). For the present research, these three advantages translate into the ability to pinpoint different activation patterns before, during, and after either time or money is primed; to detect processes that operate outside of participants’ awareness; and to differentiate the processes underlying the two mindsets.

In this research, we replicate prior behavioral research (Mogilner and Aaker, 2009) by showing that merely mentioning” time” (compared to merely mentioning “money”) leads participants to more positively evaluate a product. More importantly, we provide novel insight into the neurophysiological underpinnings of time versus money by showing that these positive product evaluations in the time condition (as compared to the money condition) are preceded by increased activation in the insula. The insula (also referred to as the insular cortex or the insular lobe) is a brain area that has been found to be a crucial mechanism in diverse but related psychological phenomena such as urging and addiction (Naqvi and Bechara, 2009), loss aversion (Knutson and Bossaerts, 2007; Knutson et al., 2007), interpersonal love (Bartels and Zeki, 2000, 2004; Beauregard et al., 2009), and brand love (Reimann et al., 2012). These functions are conceptually closely related to Mogilner and Aaker’s (2009) notion of a time-versus-money effect, which argues in favor of a greater personal connection between consumer and product right after time primes than right after money primes.

In the next section, we present the results of a content analysis on both the neurophysiological correlates and the psychological functions that underlie the concepts of time and money. Following this content analysis, we present an fMRI experiment in which participants engage in a behavioral product-rating task while their blood-oxygen level-dependent (BOLD) responses are recorded.

### The neurophysiological bases of time and money

The majority of studies that investigated the neurophysiological underpinnings of the concept of time have focused on time perception and internal time duration measurement. Using the keywords “fMRI,” “time,” “time perception,” “time psychology,” and “internal clock” to identify relevant studies. Table 1 summarizes the results of a number of fMRI studies in which brain areas were identified for specific time-related functions. For example, previous investigations provided initial evidence for an association between time perception and increased activation of the insula (Craig, 2009; Wittmann, 2009; Wittmann and van Wassenhove, 2009; Wittmann et al., 2010; van Wassenhove et al., 2011). Other studies have identified several different brain areas in which time duration measurement may be processed, including the posterior parietal cortex (Bueti et al., 2008), the prefrontal cortex (Rubia and Smith, 2004; Lewis and Miall, 2006), and the fronto-striatal circuits (Harrington et al., 2004; Hinton and Meck, 2004). In summary, the concept of time and time-related phenomena (e.g., time perception) have been associated with activation changes in the prefrontal cortex (we identified ten studies); the insula, parietal cortex, and putamen (five studies each); the caudate, frontal gyrus, operculum, striatum, and temporal gyrus (three studies each); the parietal lobule and the supplementary motor area (two studies each) as well as the cingulate cortex, cerebellum, declive, hippocampus, intraparietal sulcus, orbitofrontal cortex, parahippocampus, precuneus, semilunar lobule, sensorimotor cortex, supra-marginal gyrus, and thalamus (one study each).

**Table 1:** **Brain areas linked to the concept of time**.

Selected brain areas	Author(s) (year)	Method	N	Focal topic	Result
Caudate	Harrington et al. (2004)	Time perception task (discrimination), fMRI	24	Timing, memory, interval encoding, decision-making	Proposes that systems mediating interval encoding and decision processes are independent
	Hinton and Meck (2004)	Timing task, fMRI	6	Time perception, interval timing	Shows involvement of the frontal–striatal circuitry in human interval timing
	Jantzen et al. (2005)	Self-paced rhythmic timing task, fMRI	12	Stimulus modality and coordination pattern in rhythmic timing	Provides evidence that time and timing are served by a context-dependent distributed network rooted in basic sensorimotor processes
Cerebellum	Harrington et al. (2004)	Time perception task (discrimination), fMRI	24	Timing, memory, interval encoding, decision-making	Proposes that systems mediating interval encoding and decision processes are independent
Cingulate cortex	Hinton and Meck (2004)	Timing task, fMRI	6	Time perception, interval timing	Shows involvement of the frontal–striatal circuitry in human interval timing
Declive	Jantzen et al. (2005)	Self-paced rhythmic timing task, fMRI	12	Stimulus modality and coordination pattern in rhythmic timing	Provides evidence that time and timing are served by a context-dependent distributed network rooted in basic sensorimotor processes
Frontal gyrus	Hinton and Meck (2004)	Timing task, fMRI	6	Time perception, interval timing	Shows involvement of the frontal–striatal circuitry in human interval timing
	Jantzen et al. (2005)	Self-paced rhythmic timing task, fMRI	12	Stimulus modality and coordination pattern in rhythmic timing	Provides evidence that time and timing are served by a context-dependent distributed network rooted in basic sensorimotor processes
	Livesey et al. (2007)	Time discrimination task, fMRI	10	Time duration discrimination	Suggests that the extent of the timing “network” is overestimated, only three small brain regions certain to be directly concerned with duration judgments
Hippocampus	Harrington et al. (2004)	Time perception task (discrimination), fMRI	24	Timing, memory, interval encoding, decision-making	Proposes that systems mediating interval encoding and decision processes are independent
Insula	Lewis and Miall (2003)	Judging duration of stimuli task, fMRI	8	Time perception, neural clock	Suggests a variety of brain regions used for the measurement of both sub- and supra-second temporal durations
	Lewis and Miall (2006)	Cognitive timing task, fMRI	8	Time perception, time measurement	Provides insight into the possible role of several brain regions in attentional processing and working memory during cognitive time measurement tasks
	Livesey et al. (2007)	Time discrimination task, fMRI	10	Time duration discrimination	Suggests that the extent of the timing “network” is overestimated; only three small brain regions certain to be directly concerned with duration judgments
	Jantzen et al. (2005)	Self-paced rhythmic timing task, fMRI	12	Stimulus modality and coordination pattern in rhythmic timing	Provides evidence that time and timing are served by a context-dependent distributed network rooted in basic sensorimotor processes
	Wittmann et al. (2010)	Viewing visual events, fMRI	15	Subjective time dilation; temporal illusion	Proposes that activation of areas important for cognitive control and subjective awareness leads to temporal dilation illusion suggesting a relation of time perception and self-referential processing
Intraparietal sulcus	Schubotz et al. (2000)	Visual and auditory rhythm monitoring task, fMRI	20	Perception of temporal features of the environment	Proposes that equal brain areas responsible for time perception a planning and coordination of movements
Operculum	Schubotz et al. (2000)	Visual and auditory rhythm monitoring task, fMRI	20	Perception of temporal features of the environment	Proposes that equal brain areas responsible for time perception a planning and coordination of movements
	Coull et al. (2004)	Attention to time or color stimulus attributes task, fMRI	12	Subjective time perception, attention	Shows more accurate processing of temporal pulses throughout the stimulus duration by enhanced activity in functionally specialized brain regions due to increased time attention
	Morillon et al. (2009)	Time estimation task, fMRI	17	Perception of time	Proposes a three-staged model of time estimation with a duplicated collating process and unique counting
Orbitofrontal cortex	Hinton and Meck (2004)	Timing task, fMRI	6	Time perception, interval timing	Shows involvement of the frontal–striatal circuitry in human interval timing
Parahippocampus	Harrington et al. (2004)	Time perception task (discrimination), fMRI	24	Timing, memory, interval encoding, decision-making	Proposes that systems mediating interval encoding and decision processes are independent
Parietal cortex	Lewis and Miall (2003)	Judging duration of stimuli task, fMRI	8	Time perception, neural clock	Suggests a variety of brain regions used for the measurement of both sub- and supra-second temporal durations
	Pastor et al. (2004)	Time discrimination task, fMRI	14	Time discrimination	Proposes that the frontal brain areas play a key role in temporal processing of somatosensory events
	Harrington et al. (2004)	Time perception task (discrimination), fMRI	24	Timing, memory, interval encoding, decision-making	Proposes that systems mediating interval encoding and decision processes are independent
	Jantzen et al. (2005)	Self-paced rhythmic timing task, fMRI	12	Stimulus modality and coordination pattern in rhythmic timing	Provides evidence that time and timing are served by a context-dependent distributed network rooted in basic sensorimotor processes
	Morillon et al. (2009)	Time estimation task, fMRI	17	Perception of time	Proposes a three-staged model of time estimation with a duplicated collating process and unique counting
Parietal lobule	Pastor et al. (2004)	Time discrimination task, fMRI	14	Time discrimination	Proposes that the frontal brain areas play a key role in temporal processing of somatosensory events
	Jantzen et al. (2005)	Self-paced rhythmic timing task, fMRI	12	Stimulus modality and coordination pattern in rhythmic timing	Provides evidence that time and timing are served by a context-dependent distributed network rooted in basic sensorimotor processes
Precuneus	Harrington et al. (2004)	Time perception task (discrimination), fMRI	24	Timing, memory, interval encoding, decision-making	Proposes that systems mediating interval encoding and decision processes are independent
Prefrontal cortex	Lewis and Miall (2003)	Judging duration of stimuli task, fMRI	8	Time perception, neural clock	Suggests a variety of brain regions used for the measurement of both sub- and supra-second temporal durations
	Hinton and Meck (2004)	Timing task, fMRI	6	Time perception, interval timing	Shows involvement of the frontal–striatal circuitry in human interval timing
	Lewis and Miall (2006)	Cognitive timing task, fMRI	8	Time perception, time measurement	Provides insight into the possible role of several brain regions in attentional processing and working memory during cognitive time measurement tasks
	Vallesi et al. (2009)	Visual discrimination task/fMRI	14	Time processing	Shows the critical role of the right dorsolateral prefrontal cortex to observe the strategically mediated behavioral effects in the variable foreperiod paradigm
	Morillon et al. (2009)	Time estimation task, fMRI	17	Perception of time	Proposes a three-staged model of time estimation with a duplicated collating process and unique counting
Premotor cortex	Schubotz et al. (2000)	Visual and auditory rhythm monitoring tasks, fMRI	20	Perception of temporal features of the environment	Proposes that equal brain areas responsible for time perception a planning and coordination of movements
	Lewis and Miall (2003)	Judging duration of stimuli task, fMRI	8	Time perception, neural clock	Suggests a variety of brain regions used for the measurement of both sub- and supra-second temporal durations
	Coull et al. (2004)	Attention to time or color stimulus attributes task, fMRI	12	Subjective time perception, attention	Shows more accurate processing of temporal pulses throughout the stimulus duration by enhanced activity in functionally specialized brain regions due to increased time attention
	Pastor et al. (2004)	Time discrimination task, fMRI	14	Time discrimination	Proposes that the frontal brain areas play a key role in temporal processing of somatosensory events
	Pouthas et al. (2005)	Long and short duration estimation task, fMRI	6	Time perception, interval estimation	Proposes the support of several brain areas to a clock mechanism, decision and response-related processes, and active maintenance of temporal information
Putamen	Schubotz et al. (2000)	Visual and auditory rhythm monitoring task, fMRI	20	Perception of temporal features of the environment	Proposes that equal brain areas responsible for time perception a planning and coordination of movements
	Nenadic et al. (2003)	Perceptual timing task, fMRI	15	Time perception, internal clock	Proposes an interaction between brain areas with modality-dependent sensory cortical, timing-specific, and attention and memory function
	Hinton and Meck (2004)	Timing task, fMRI	6	Time perception, interval timing	Shows involvement of the frontal–striatal circuitry in human interval timing
	Jantzen et al. (2005)	Self-paced rhythmic timing task, fMRI	12	Stimulus modality and coordination pattern in rhythmic timing	Provides evidence that time and timing are served by a context-dependent distributed network rooted in basic sensorimotor processes
	Livesey et al. (2007)	Time discrimination task, fMRI	10	Time duration discrimination	Suggests that the extent of the timing “network” is overestimated, only three small brain regions certain to be directly concerned with duration judgments
Semilunar lobule	Schubotz et al. (2000)	Visual and auditory rhythm monitoring task, fMRI	20	Perception of temporal features of the environment	Proposes that equal brain areas responsible for time perception a planning and coordination of movements
Sensorimotor cortex	Jantzen et al. (2005)	Self-paced rhythmic timing task, fMRI	12	Stimulus modality and coordination pattern in rhythmic timing	Provides evidence that time and timing are served by a context-dependent distributed network rooted in basic sensorimotor processes
Striatum	Schubotz et al. (2000)	Visual and auditory rhythm monitoring tasks, fMRI	20	Perception of temporal features of the environment	Proposes that equal brain areas responsible for time perception a planning and coordination of movements
	Nenadic et al. (2003)	Perceptual timing task, fMRI	15	Time perception, internal clock	Proposes an interaction between brain areas with modality-dependent sensory cortical, timing-specific, and attention and memory function
	Coull et al. (2004)	Attention to time or color stimulus attributes task, fMRI	12	Subjective time perception, attention	Shows more accurate processing of temporal pulses throughout the stimulus duration by enhanced activity in functionally specialized brain regions due to increased time attention
Supplementary motor area	Schubotz et al. (2000)	Visual and auditory rhythm monitoring task, fMRI	20	Perception of temporal features of the environment	Proposes that equal brain areas responsible for time perception a planning and coordination of movements
	Jantzen et al. (2005)	Self-paced rhythmic timing task, fMRI	12	Stimulus modality and coordination pattern in rhythmic timing	Provides evidence that time and timing are served by a context-dependent distributed network rooted in basic sensorimotor processes
Supra-marginal gyrus	Livesey et al. (2007)	Time discrimination task, fMRI	10	Time duration discrimination	Suggests that the extent of the timing “network” is overestimated; only three small brain regions certain to be directly concerned with duration judgments
Thalamus	Hinton and Meck (2004)	Timing task, fMRI	6	Time perception, interval timing	Shows involvement of the frontal–striatal circuitry in human interval timing
Temporal gyrus	Hinton and Meck (2004)	Timing task, fMRI	6	Time perception, interval timing	Shows involvement of the frontal–striatal circuitry in human interval timing
	Jantzen et al. (2005)	Self-paced rhythmic timing task, fMRI	12	Stimulus modality and coordination pattern in rhythmic timing	Provides evidence that time and timing are served by a context-dependent distributed network rooted in basic sensorimotor processes
	Morillon et al. (2009)	Time estimation task, fMRI	17	Perception of time	Proposes a three-staged model of time estimation with a duplicated collating process and unique counting
Temporal sulcus	Morillon et al. (2009)	Time estimation task, fMRI	17	Perception of time	Proposes a three-staged model of time estimation with a duplicated collating process and unique counting

*Studies were sorted alphabetically by brain area and then chronologically within each brain area*.

The neurophysiological underpinnings of the concept of money have been subject to far more studies than those of time. Using the keywords “fMRI,” “money,” “money perception,” and “money psychology” to locate relevant studies. Table 2 summarizes the results of several fMRI studies in which brain areas were identified for money-related functions. For example, it was found that the mere anticipation of monetary gains activates the ventral and dorsal striatum, anterior thalamus, anterior insula, cortical motor regions, and the cerebellar vermis (Knutson et al., 2003). Furthermore, the ventral striatum and the insula have been implicated mainly in the processing of concrete monetary rewards (Kuhnen and Knutson, 2005). Another investigation revealed that (1) fronto-parietal regions (i.e., regions of the lateral prefrontal cortex and posterior parietal cortex) elicit greater activation for delayed monetary rewards, (2) limbic and paralimbic cortical structures (i.e., the ventral striatum, medial prefrontal cortex, and posterior cingulate cortex) reveal greater activation for immediately available rewards, and (3) that fronto-parietal regions show greater activation for both immediate and delayed monetary rewards (McClure et al., 2004). In summary, the concept of money and money-related phenomena (e.g., monetary reward) have previously mainly been related to activation changes in the prefrontal cortex (we identified eight studies); the cingulate cortex, nucleus accumbens (seven studies each); the insula, striatum, and thalamus (six studies each); the amygdala, dorsal caudate, and frontal cortex (five studies each); the orbitofrontal cortex (four studies); the midbrain and putamen (three studies each); the frontal gyrus, globus pallidus, parietal lobule, and precuneus (two studies each) as well as the cerebellar vermis, cerebellum, frontal pole, fusiform gyrus, hippocampus, hypothalamus, operculum, medial temporal lobe, motor cortex, orbital gyrus, and the precentral gyrus (one study each).

**Table 2:** **Brain areas linked to the concept of money**.

Selected brain areas	Author(s) (year)	Method	N	Focal topic	Result
Amygdala	Breiter et al. (2001)	Gambling task, fMRI	12	Functional dissociation of experience and anticipation of rewards	Shows that responses to prospects and outcomes were generally, seen in the same regions. A common circuitry to the processing of diverse rewards is suggested
	Knutson et al. (2001b)	Monetary incentive delay task, fMRI	9	Functional dissociation of reward anticipation and outcome	Shows differential recruitment of regions along the trajectory of ascending dopamine projections in reward anticipation and outcomes
	Elliott et al. (2003)	Rewarded target detection task, fMRI	12	Functional distinction of value and magnitude	Shows different response of brain regions to the reward’s value suggesting functional distinction in response patterns within a distributed reward system
	Fujiwara et al. (2009)	Free-choice task, fMRI	17	Cingulate activations for different levels of monetary gain and loss	Provides evidence for separate and common coding, but not separate structures, of monetary reward and punishment in distinct brain regions
	Spreckelmeyer et al. (2009)	Incentive delay task, fMRI	32	Men/women; monetary versus social reward	Proposes that neural structures constituting the human reward system are proportionally activated for increasing levels of reward, independent of incentive type
Caudate	Elliott et al. (2000)	Monetary gambling task, fMRI	9	Functional dissociation of gains and losses	Shows dissociable neural responses to rewards and penalties dependent on the experienced psychological context.
	Knutson et al. (2000a)	Monetary incentive delay task, fMRI	12	Functional dissociation of reward and punishment	Shows similar activation patterns in reward and punishment trials, but differences in a group analyses
	Knutson et al. (2001a)	Monetary incentive task, fMRI	8	Functional dissociation of anticipation and outcome	Provides evidence that striatal areas code for expected incentive magnitude, a region in the nucleus accumbens codes for expected positive incentive value
	Knutson et al. (2001b)	Monetary incentive delay task, fMRI	9	Functional dissociation of reward anticipation and outcome	Shows differential recruitment of regions along the trajectory of ascending dopamine projections in reward anticipation and outcomes
	Spreckelmeyer et al. (2009)	Incentive delay task, fMRI	32	Men/women; monetary versus social reward	Proposes that neural structures constituting the human reward system are proportionally activated for increasing levels of reward, independent of incentive type
Cerebellar vermis	Knutson et al. (2001b)	Monetary incentive delay task, fMRI	9	Functional dissociation of reward anticipation and outcome	Shows differential recruitment of regions along the trajectory of ascending dopamine projections in reward anticipation and outcomes
Cerebellum	Fujiwara et al. (2009)	Free-choice task, fMRI	17	Cingulate activations for different levels of monetary gain and loss	Provides evidence for separate and common coding, but not separate structures, of monetary reward and punishment in distinct brain regions
Cingulate cortex	Elliott et al. (2000)	Monetary gambling task, fMRI	9	Functional dissociation of gains and losses	Shows dissociable neural responses to rewards and penalties dependent on the experienced psychological context
	Knutson et al. (2000a)	Monetary incentive delay task, fMRI	12	Functional dissociation of reward and punishment	Shows similar activation patterns in reward and punishment trials, but differences in a group analyses
	Knutson et al. (2001a)	Monetary incentive task, fMRI	8	Functional dissociation of anticipation and outcome	Provides evidence that striatal areas code for expected incentive magnitude, a region in the nucleus accumbens codes for expected positive incentive value
	Kirsch et al. (2003)	Rewarded reaction time task, fMRI	27	Motivational value of money	Proposes that anticipation of a monetary reward produced stronger activation than the anticipation of positive verbal feedback due to motivation-dependent reactivity of the brain reward system
	Knutson et al. (2003)	Monetary incentive delay task, fMRI	12	Functional dissociation of gains and losses	Suggests that in the context of processing monetary rewards, a region of the medial frontal cortex preferentially tracks rewarding outcomes
	Nieuwenhuis et al. (2005)	Monetary gambling task, fMRI	14	Range sensitivity	Shows that activity in the reward-sensitive brain areas is highly sensitive to the range of possible outcomes. Suggesting context-dependency of the brain’s scaling of motivational value of events
	Fujiwara et al. (2009)	Free-choice task, fMRI	17	Cingulate activations for different levels of monetary gain and loss	Provides evidence for separate and common coding, but not separate structures, of monetary reward and punishment in distinct brain regions
Frontal cortex	Knutson et al. (2000a)	Monetary incentive delay task, fMRI	12	Functional dissociation of reward and punishment	Shows similar activation patterns in reward and punishment trials, but differences in a group analyses
	Knutson et al. (2003)	Monetary incentive delay task, fMRI	12	Functional dissociation of gains and losses	Suggests that in the context of processing monetary rewards, a region of the medial frontal cortex preferentially tracks rewarding outcomes
	Hampton et al. (2006)	Decision-making task, fMRI	16	Monetary decision-making	Suggests that key decision-making brain regions use an abstract model of task structure, based on higher-order structure rather simple reinforcement learning, to guide behavioral choice
	Kim et al. (2006)	Instrumental choice task, fMRI	16	Avoidance learning, reinforcements	Shows that neural activity in the medial orbitofrontal cortex increased following the receipt of reward and the successful avoidance of an aversive outcome, thereby serving to reinforce actions during instrumental avoidance
	Kable and Glimcher (2007)	Intertemporal choice task, fMRI	10	Monetary decision-making, subjective choice	Suggests that the subjective value of potential rewards is explicitly represented in the human brain
Frontal gyrus	Fujiwara et al. (2009)	Free-choice task, fMRI	17	Cingulate activations for different levels of monetary gain and loss	Provides evidence for separate and common coding, but not separate structures, of monetary reward and punishment in distinct brain regions
	Becchio et al. (2011)	Judgment task, fMRI	20	Neuronal representation of the functional use of money.	Shows that destruction of money elicits activation within the same temporo-parietal network which is associated with the knowledge of the functional use of concrete tools
Frontal pole	Knutson et al. (2001a)	Monetary incentive task, fMRI	8	Functional dissociation of anticipation and outcome	Provides evidence that striatal areas code for expected incentive magnitude, a region in the nucleus accumbens codes for expected positive incentive value
Fusiform gyrus	Becchio et al. (2011)	Judgment task, fMRI	20	Neuronal representation of the functional use of money.	Shows that destruction of money elicits activation within the same temporo-parietal network, which is associated with the knowledge of the functional use of concrete tools
Globus pallidus	Elliott et al. (2000)	Monetary gambling task, fMRI	9	Functional dissociation of gains and losses	Results revealed dissociable neural responses to rewards and penalties that were dependent on the psychological context in which they were experienced
	Kirsch et al. (2003)	Rewarded reaction time task, fMRI	27	Motivational value of money	Proposes that anticipation of a monetary reward produced stronger activation than the anticipation of positive verbal feedback due to motivation-dependent reactivity of the brain reward system
Hippocampus	Elliott et al. (2000)	Monetary gambling task, fMRI	9	Functional dissociation of gains and losses	Shows dissociable neural responses to rewards and penalties dependent on the experienced psychological context
Hypothalamus	Breiter et al. (2001)	Gambling task, fMRI	12	Functional dissociation of experience and anticipation of rewards	Shows that responses to prospects and outcomes were generally, seen in the same regions. A common circuitry to the processing of diverse rewards is suggested
Insula	Elliott et al. (2000)	Monetary gambling task, fMRI	9	Functional dissociation of gains and losses	Shows dissociable neural responses to rewards and penalties dependent on the experienced psychological context
	Knutson et al. (2000a)	Monetary incentive delay task, fMRI	12	Functional dissociation of reward and punishment	Shows similar activation patterns in reward and punishment trials, but differences in a group analyses
	Knutson et al. (2001b)	Monetary incentive delay task, fMRI	9	Functional dissociation of reward anticipation and outcome	Shows differential recruitment of regions along the trajectory of ascending dopamine projections in reward anticipation and outcomes
	Knutson et al. (2003)	Monetary incentive delay task, fMRI	12	Functional dissociation of gains and losses	Suggests that in the context of processing monetary rewards, a region of the medial frontal cortex preferentially tracks rewarding outcomes
	Knutson et al. (2008)	Savings hold or purchase (SHOP) task, fMRI	24	Neural antecedents of the endowment effect	Shows greater nucleus accumbens activation for preferred products across buy and sell conditions combined, greater mesial prefrontal cortex activation in response to low prices (buying versus selling). Right insular activation for preferred products (selling) predicted individual differences in susceptibility to the endowment effect
	Fujiwara et al. (2009)	Free-choice task, fMRI	17	Cingulate activations for different levels of monetary gain and loss	Provides evidence for separate and common coding, but not separate structures, of monetary reward and punishment in distinct brain regions
Midbrain	Elliott et al. (2000)	Monetary gambling task, fMRI	9	Functional dissociation of gains and losses	Shows dissociable neural responses to rewards and penalties dependent on the experienced psychological context
	Fujiwara et al. (2009)	Free-choice task, fMRI	17	Cingulate activations for different levels of monetary gain and loss	Provides evidence for separate and common coding, but not separate structures, of monetary reward and punishment in distinct brain regions
	Mobbs et al. (2009)	Reward-pursuit task, fMRI	14	Reduced performance for larger-than-average rewards	Shows that activation of the ventral midbrain correlates with reduced number of captures and increased number of near-misses associated with imminent high rewards relating to choking under pressure and “overmotivation”
Motor cortex	Knutson et al. (2001b)	Monetary incentive delay task, fMRI	9	Functional dissociation of reward anticipation and outcome	Shows differential recruitment of regions along the trajectory of ascending dopamine projections in reward anticipation and outcomes
Nucleus accumbens	Knutson et al. (2000b)	Monetary incentive delay tasks, fMRI	12	Anticipation of increasing rewards	Demonstrates nucleus accumbens activity during reward anticipation indicating a key role in generating the experience of positive affect
	Breiter et al. (2001)	Gambling task, fMRI	12	Functional dissociation of experience and anticipation of rewards	Shows that responses to prospects and outcomes were generally, seen in the same regions. A common circuitry to the processing of diverse rewards is suggested
	Knutson et al. (2001b)	Monetary incentive delay task, fMRI	9	Functional dissociation of reward anticipation and outcome	Shows differential recruitment of regions along the trajectory of ascending dopamine projections in reward anticipation and outcomes
	Kirsch et al. (2003)	Rewarded reaction time task, fMRI	27	Motivational value of money	Proposes that anticipation of a monetary reward produced stronger activation than the anticipation of positive verbal feedback due to motivation-dependent reactivity of the brain reward system.
	Cooper and Knutson (2008)	Monetary incentive task, fMRI	12	Certain/uncertain outcomes; salience/valence	Suggests that in the nucleus accumbens different activation increases and decreases for gains and losses under outcome (un-)certainty, separately represent both valence and salience following appetitive motivation.
	Knutson et al. (2008)	Savings hold or purchase (SHOP) task, fMRI	24	Neural antecedents of the endowment effect	Shows greater nucleus accumbens activation for preferred products across buy and sell conditions combined, greater medial prefrontal cortex activation in response to low prices (buying versus selling). Right insular activation for preferred products (selling) predicted individual differences in susceptibility to the endowment effect
	Spreckelmeyer et al. (2009)	Incentive delay task, fMRI	32	Men/women; monetary versus social reward	Proposes that neural structures constituting the human reward system are proportionally activated for increasing levels of reward, independent of incentive type
Operculum	Fujiwara et al. (2009)	Free-choice task, fMRI	17	Cingulate activations for different levels of monetary gain and loss	Provides evidence for separate and common coding, but not separate structures, of monetary reward and punishment in distinct brain regions
Orbital gyrus	Breiter et al. (2001)	Gambling task, fMRI	12	Functional dissociation of experience and anticipation of rewards	Shows that responses to prospects and outcomes were generally, seen in the same regions. A common circuitry to the processing of diverse rewards is suggested
Orbitofrontal cortex	O’Doherty et al. (2001)	Emotion-related visual reversal-learning task, fMRI	6	Functional dissociation of gains and losses	Shows that the magnitude of the brain activation correlated with the magnitude of the abstract rewards and punishments (gaining or losing money) received through emotional involvement
	Elliott et al. (2003)	Rewarded target detection task, fMRI	12	Functional distinction of value and magnitude	Shows different response of brain regions to the reward’s value suggesting functional distinction in response patterns within a distributed reward system
	Kirsch et al. (2003)	Rewarded reaction time task, fMRI	27	Motivational value of money	Proposes that anticipation of a monetary reward produced stronger activation than the anticipation of positive verbal feedback due to motivation-dependent reactivity of the brain reward system
	Plassmann et al. (2007)	Bidding task, fMRI	19	Willingness-to-pay, cognitive computation of financial resources	Provides evidence that activity in the medial orbitofrontal cortex and in the dorsolateral prefrontal cortex encodes subjects’ willingness-to-pay suggesting that the medial orbitofrontal cortex encodes the value of goals in decision-making
	Fujiwara et al. (2009)	Free-choice task, fMRI	17	Cingulate activations for different levels of monetary gain and loss	Separate and common coding of monetary reward and punishment indistinct subregions of the cingulate cortex. However, the study does not support separate structures because same key regions were activated for both gains and losses
Parietal cortex	Knutson et al. (2001a)	Monetary incentive task, fMRI	9	Functional dissociation of anticipation and outcome	Provides evidence that striatal areas code for expected incentive magnitude, a region in the nucleus accumbens codes for expected positive incentive value
	Knutson et al. (2003)	Monetary incentive delay task, fMRI	12	Functional dissociation of gains and losses	Suggests that in the context of processing monetary rewards, a region of the medial frontal cortex preferentially tracks rewarding outcomes
Parietal lobule	Nieuwenhuis et al. (2005)	Monetary gambling task, fMRI	14	Range sensitivity	Shows that activity in the reward-sensitive brain areas is highly sensitive to the range of possible outcomes. Suggesting context-dependency of the brain’s scaling of motivational value of events
	Fujiwara et al. (2009)	Free-choice task, fMRI	17	Cingulate activations for different levels of monetary gain and loss	Provides evidence for separate and common coding, but not separate structures, of monetary reward and punishment in distinct brain regions
Precentralgyrus	Becchio et al. (2011)	Judgment task, fMRI	20	Neuronal representation of the functional use of money.	Shows that destruction of money elicits activation within the same temporo-parietal network, which is associated with the knowledge of the functional use of concrete tools
Precuneus	Spreckelmeyer et al. (2009)	Incentive delay task, fMRI	32	Men/women; monetary versus social reward	Proposes that neural structures constituting the human reward system are proportionally activated for increasing levels of reward, independent of incentive type
	Becchio et al. (2011)	Judgment task, fMRI	20	Neuronal representation of the functional use of money.	Shows that destruction of money elicits activation within the same temporo-parietal network, which is associated with the knowledge of the functional use of concrete tools
Prefrontal cortex	Elliott et al. (2000)	Monetary gambling task, fMRI	9	Functional dissociation of gains and losses	Shows dissociable neural responses to rewards and penalties dependent on the experienced psychological context
	Knutson et al. (2001a)	Monetary incentive task, fMRI	8	Functional dissociation of anticipation and outcome	Provides evidence that striatal areas code for expected incentive magnitude, a region in the nucleus accumbens codes for expected positive incentive value
	Nieuwenhuis et al. (2005)	Monetary gambling task, fMRI	14	Range sensitivity	Shows that activity in the reward-sensitive brain areas is highly sensitive to the range of possible outcomes. Suggesting context-dependency of the brain’s scaling of motivational value of events
	Daw et al. (2006)	Gambling task, fMRI	14	Exploration-exploitation-dilemma	Proposes that different brain regions are active during exploratory and exploitative decision-making suggesting action selection under uncertainty that involves switching between exploratory and exploitative behavioral modes
	Knutson et al. (2008)	Savings hold or purchase (SHOP) task, fMRI	24	Neural antecedents of the endowment effect	Shows greater nucleus accumbens activation for preferred products across buy and sell conditions combined, greater mesial prefrontal cortex activation in response to low prices (buying versus selling). Right insular activation for preferred products (selling) predicted individual differences in susceptibility to the endowment effect
	Hampton et al. (2006)	Decision-making task, fMRI	16	Higher-order decision-making	Suggests that key decision-making brain regions use an abstract model of task structure, based on higher-order structure rather simple reinforcement learning, to guide behavioral choice
	Fujiwara et al. (2009)	Free-choice task, fMRI	17	Cingulate activations for different levels of monetary gain and loss	Provides evidence for separate and common coding, but not separate structures, of monetary reward and punishment in distinct brain regions
	Weber et al. (2009)	Estimation task, fMRI	24	Money illusion	Proposes that ventromedial prefrontal cortex exhibited money illusion
Premotor cortex	Elliott et al. (2003)	Rewarded target detection task, fMRI	12	Functional distinction of value and magnitude	Shows different response of brain regions to the reward’s value suggesting functional distinction in response patterns within a distributed reward system
Putamen	Knutson et al. (2000a)	Monetary incentive delay task, fMRI	12	Functional dissociation of reward and punishment	Shows similar activation patterns in reward and punishment trials, but differences in a group analyses
	Knutson et al. (2001b)	Monetary incentive delay task, fMRI	9	Functional dissociation of reward anticipation and outcome	Shows differential recruitment of regions along the trajectory of ascending dopamine projections in reward anticipation and outcomes
	Kirsch et al. (2003)	Rewarded reaction time task, fMRI	27	Motivational value of money	Proposes that anticipation of a monetary reward produced stronger activation than the anticipation of positive verbal feedback due to motivation-dependent reactivity of the brain reward system
Striatum	Delgado et al. (2000)	Monetary gambling task	9	Functional dissociation of gains and losses	Shows that activation in different brain regions was sustained following a reward feedback, but decreased below baseline following a punishment feedback
	Elliott et al. (2000)	Monetary gambling task, fMRI	9	Functional dissociation of gains and losses	Shows dissociable neural responses to rewards and penalties dependent on the experienced psychological context
	Elliott et al. (2003)	Rewarded target detection task, fMRI	12	Functional distinction of value and magnitude	Shows different response of brain regions to the reward’s value suggesting functional distinction in response patterns within a distributed reward system
	Knutson et al. (2003)	Monetary incentive delay task, fMRI	12	Functional dissociation of gains and losses	Suggests that in the context of processing monetary rewards, a region of the medial frontal cortex preferentially tracks rewarding outcomes
	Nieuwenhuis et al. (2005)	Monetary gambling task, fMRI	14	Range sensitivity, value and magnitude	Shows that activity in the reward-sensitive brain areas is highly sensitive to the range of possible outcomes. Suggesting context-dependency of the brain’s scaling of motivational value of events
	Fujiwara et al. (2009)	Free-choice task, fMRI	17	Cingulate activations for different levels of monetary gain and loss	Provides evidence for separate and common coding, but not separate structures, of monetary reward and punishment in distinct brain regions
Temporal gyrus	Fujiwara et al. (2009)	Free-choice task, fMRI	17	Cingulate activations for different levels of monetary gain and loss	Provides evidence for separate and common coding, but not separate structures, of monetary reward and punishment in distinct brain regions
Thalamus	Elliott et al. (2000)	Monetary gambling task, fMRI	9	Functional dissociation of gains and losses	Results revealed dissociable neural responses to rewards and penalties that are dependent on the psychological context in which they are experienced
	Knutson et al. (2000a)	Monetary incentive delay task, fMRI	12	Functional dissociation of reward and punishment	Shows similar activation patterns in reward and punishment trials, but differences in a group analyses
	Knutson et al. (2001b)	Monetary incentive delay task, fMRI	9	Functional dissociation of reward anticipation and outcome	Shows differential recruitment of regions along the trajectory of ascending dopamine projections in reward anticipation and outcomes
	Kirsch et al. (2003)	Rewarded reaction time task, fMRI	27	Motivational value of money	Proposes that anticipation of a monetary reward produced stronger activation than the anticipation of positive verbal feedback due to motivation-dependent reactivity of the brain reward system
	Knutson et al. (2003)	Monetary incentive delay task, fMRI	12	Functional dissociation of gains and losses	Suggests that in the context of processing monetary rewards, a region of the medial frontal cortex preferentially tracks rewarding outcomes
	Spreckelmeyer et al. (2009)	Incentive delay task, fMRI	32	Men/women; monetary versus social reward	Proposes that neural structures constituting the human reward system are proportionally activated for increasing levels of reward, independent of incentive type

*Studies were sorted alphabetically by brain area and then chronologically within each brain area*.

While these studies provide interesting insights into the neurophysiological processes underlying either time or money, to our knowledge no previous study has directly compared the neurophysiology of time primes with that of money primes. Following prior behavioral research on the time-versus-money effect in product evaluations (Mogilner and Aaker, 2009), we would expect a greater emotional mindset for time primes than for money primes, because the concept of time seems to boost the formation and maintenance of close personal connections between consumer and product to a greater extent than a money mindset. Specific brain areas have been associated with emotional processing in prior research (Bechara and Damasio, 2005; Reimann and Bechara, 2010; Reimann and Zimbardo, 2011). The aforementioned content analysis identified several of these emotional brain areas, including the insula, the amygdala, and parts of the prefrontal cortex.

But, why should either time or money be associated with a higher degree of activation in these emotional brain regions? Both money (Dunn et al., 2008; Vohs et al., 2008) and time (Sheldon and Elliot, 1999; Mogilner, 2010) can foster well-being and elicit an emotional mindset, and can, therefore, lead to stronger urgings to recreate or maintain this state. Indeed, money can possess a drug-like character (Roll et al., 2000; Lea and Webley, 2006), which may explain why people have an urge for it. On the other hand, it has been argued that time has greater emotional meaning than money (Mogilner and Aaker, 2009) and that money even weakens the emotional link to objects as well as to people (Vohs et al., 2006, 2008; Liu and Aaker, 2008). Further, when asked to think about traveling back in time, people tend to forget about negative aspects from their past and instead remember positive situations and feelings (Carstensen et al., 2000), as if they have an urge for the “good old times.” As such, when primed with time (versus money), reminders of positive feelings might elicit a stronger emotional mindset, lead to a higher degree of urging to recreate or maintain this emotional state, and positively influence downstream product attitudes. Yet, it is important to note that while the present research builds on the notions of previous research (e.g., Mogilner and Aaker, 2009), it is also exploratory in nature. As such, we acknowledge that alternative arguments on whether time or money elicit greater emotional responses can be brought forward.

One brain area has been associated with the aforementioned functions – that is, urging and processing feelings of personal connectedness. For several decades, research in functional neuroanatomy has held that the insula is crucial in the integration of bodily information into emotional and motivational functions (Mesulam and Mufson, 1982). Humans perceive feelings from their bodies, which are fed into an afferent neural system that represents all aspects of the physiological condition of the physical body (Craig, 2002). For example, perceiving facial expressions ranging from sad to happy can trigger bodily responses, which in turn are associated with insula activation (Britton et al., 2006). Subsequently, the insula integrates these bodily states into conscious feelings and decision-making processes (Bechara and Damasio, 2005; Reimann and Bechara, 2010; Reimann and Zimbardo, 2011). The insula has also been shown a crucial brain region in urging and addiction (Naqvi and Bechara, 2009) as well as – in more applied domains – in loss aversion (Knutson and Bossaerts, 2007; Knutson et al., 2007), interpersonal love (Bartels and Zeki, 2000, 2004; Beauregard et al., 2009), and brand love (Reimann et al., 2012).

These investigations provide compelling evidence on certain insula functions that conceptually map the psychological functions of the time-versus-money effect. As an important word of caution, however, we acknowledge that like most prior cognitive neuroscience research, the present study relies on reverse inference in that activation of a particular brain area (insula) is interpreted as support for engagement of particular psychological processes (urging, personal connection). In dealing with this issue, we followed the recommendations by Poldrack (2006) and reported task characteristics and showed replication of prior behavioral evidence. Yet, we recognize that the breath of functions associated with the insula leave room for interpretation. As such, one can only hold the insula responsible for its most basic function – that is, integration of bodily information into emotional and motivational functions (Mesulam and Mufson, 1982) beyond which the particular psychological process becomes less clear.

## Materials and Methods

The present experiment investigates the question of whether the activation of the concept of either time or money leads to distinct neurophysiological responses, which in turn may help to explain behavioral differences in how consumers evaluate products. On the basis of Mogilner and Aaker’s (2009) research, we designed a behavioral product-rating task in which participants engaged in product evaluations while undergoing fMRI.

### Participants

Forty-four right-handed, healthy subjects (23 females; *M*_age_ =24.8 years, SD_age_ = 4.0 years; ranging from 20 to 44 years) participated in the study for a compensation of 15 euro. Participants were recruited from the neuroscience subject pool of a public university. The study was approved by the university’s ethics committee, participants were screened for medical eligibility, and written informed consent was obtained from each participant prior to the experiment. Because we focused our analyses on a specific product (i.e., participants’ wristwatch), subjects were also asked whether they had bought their current wristwatch themselves. Those participants that confirmed having bought their watch themselves were selected for the study, asked to take a picture of their watch, and send it to us. Each picture was taken with the watch in the center in front of a neutral background. Participants were randomly assigned to one of two conditions in this between-subject experimental design. In one condition, participants were primed with the concept of time; in the other condition, participants were primed with the concept of money. One participant was excluded from subsequent data analyses because of extensive head motion during the brain scan. It is important to note that we used the same procedure not only for the wristwatch, on which we focused our subsequent analyses, but also for three other products (i.e., mobile phone, MP3 player, and laptop computer).

### Scan preparation and behavioral task

Before entering the brain scanner, participants underwent a short training version of the task to alleviate task-related confusion. Next, participants received the initial prime. We employed two established priming techniques (Strack et al., 1985; Dunn and Schweitzer, 2005; Lee et al., 2009), one outside the brain scanner and one inside the brain scanner. Outside the brain scanner, participants were given 5 min to write about anything that came to their minds when thinking about one of the two concepts. Before participants were placed inside the scanner, we ensured that all subjects were clear about what they were asked to do and what they were asked to think of. That is, in the money condition, we ensured participants had thought about the amount spent on their wristwatch, and in the time condition, we ensured participants had thought about the time span they had owned their wristwatch. Inside the brain scanner, word primes that aimed at inducing one mindset or the other were given visually (Burnham, 2000; Bargh et al., 2001; Mogilner et al., 2008; Mogilner and Aaker, 2009). Participants were each placed supine inside a full-body 3.0 T Siemens Magnetom Trio scanner (manufactured by Siemens AG in Erlangen, Germany) fitted with a 12-channel matrix head coil. Participants were presented with the full version of the product-rating task while resting on their backs. Task stimuli were projected into the scanner; participants could see the stimuli in a mirror located directly before their eyes. The task consisted of 15 trials with five phases each to generate a sufficient number of volumes for the neuroimaging data analyses. For presentation of the task stimuli and accurate recording of participants’ product ratings, E-Prime Professional software, version 2.0.8.74 (manufactured by Psychology Software Tools Inc. in Pittsburg, PA, USA) was used.

Each participant saw a series of seven word primes (presented for 10 s each). In the time condition, participants saw *time*, *to have time*, *win time*, *time management*, *enjoy time*, *use time*, and *time* again. In the money condition, participants were shown *money*, *to have money*, *win money*, *money management*, *enjoy money*, *use money*, and *money* again. It is important to note that we used words and phrases representing the general concepts of time and money rather than a specific amount of time or a specific monetary possession. In summary, in our study, we activated the concepts of either time or money through the use of mental priming techniques, which heightened the salience of either time or money. Thus, priming acted as a reminder of both concepts (Vohs et al., 2006; Liu and Aaker, 2008; Mogilner and Aaker, 2009).

**Figure 1:**
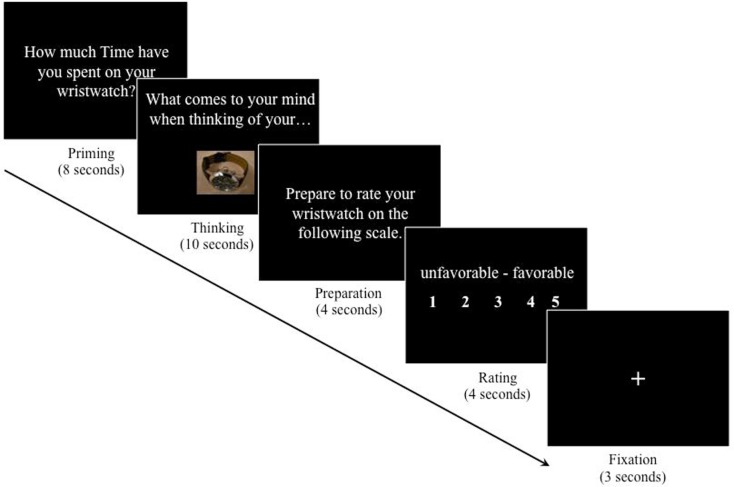
**Product-rating task**.

The initial word priming was followed by the behavioral rating task, which consisted of a repeated five-step trial (Figure 1). First, for 8 s, participants were asked either “How much Time have you spent on your wristwatch?” or “How much Money have you spent on your wristwatch?” (“priming phase”). Second, for 10 s, participants were prompted to think about the product with the question “What comes to your mind when thinking of your wristwatch?” (“thinking phase”). During this phase, participants were also shown the picture of their own wristwatch. Third, for 4 s, participants were told to prepare themselves to rate their wristwatch (“preparation phase”). Fourth, for 4 s, participants rated their wristwatch on a five-point Likert-type semantic differential scale from unfavorable to favorable by pressing one of five buttons on a response box (“rating phase”). The lowest possible rating was given with the thumb of the right hand and consecutive higher ratings were given with the next finger going to the right. Fifth and finally, a fixation cross appeared for 3 s and ended each trial (“fixation phase”) before the next trial started. The task timing was in line with previous research on mood and emotion induction (e.g., Isen et al., 1976; Isen and Gorgoglione, 1983). The trial was repeated three times. BOLD signal changes were recorded during the whole task.

### Neuroimaging data collection

We applied standard neuroimaging procedures (e.g., Reimann et al., 2010, 2011; Kable, 2011). For anatomical neuroimaging, we ran (1) a brief scan for land-marking and (2) a high-resolution whole-brain magnetization-prepared rapid gradient-echo (MPRAGE) sequence. MPRAGE sequence parameters were: echo time (TE)/repetition time (TR)/inversion time (TI) = 4.77/2,500/1,100 ms, flip angle = 7°, matrix = 256 × 256, field of view (FOV) = 256 mm, slice thickness = 1 mm without gap. For functional neuroimaging, a time series of 130 volumes with 34 slices in the sagittal plane was collected in an interleaved sequence, using single-shot gradient-echo planar imaging (TR = 2,000 ms, TE = 30 ms, flip angle = 80°, resolution = 3.5 mm × 3.5 mm × 3.5 mm, and FOV = 224 mm, 64 × 64 matrix) and allowing for whole-brain coverage in a relatively short period of time. Participants were given earplugs to reduce the distraction of scanner noise and participants’ head movements were minimized with foam pads.

### Neuroimaging data analysis

For the neuroimaging data analysis, BrainVoyager QX software, version 2.3 (manufactured by Brain Innovation B.V. in Maastricht, Netherlands) was used. A number of preprocessing steps were performed on the functional data prior to the statistical analysis. For each participant, we used standard methods of analysis (e.g., DeBettencourt et al., 2011; Hammer et al., 2011), including: (1) exclusion of the first three scans per run from the analysis to ensure that steady-state tissue magnetization was reached and, therefore, to permit T1-equilibration effects; (2) incremental linear trend removal to eliminate scanner-related signal drifts; (3) temporal high-pass filtering to remove temporal frequencies (i.e., scanner- and physiology-related noise) lower than three cycles per run; and (4) a rigid-body algorithm, which rotates and translates each functional volume in three-dimensional space in order to correct for small head movements between scans. The data was spatially smoothed with a three-dimensional Gaussian filter (i.e., 4 mm full-width at half maximum). Functional neuroimages were co-registered to the anatomical images and interpolated to cubic voxels. For anatomical orientation, the three-dimensional T1-weighted scans were used to overlay the statistical maps. To enable comparison among participants, both anatomical and functional volumes were spatially normalized into Talairach-type space (Talairach and Tournoux, 1988).

In line with prior priming and emotion induction research (e.g., Damasio et al., 2000) and because the study aimed at identifying the neurophysiological underpinnings of time versus money, we focused our analyses of the neuroimaging data on the” priming phase”; that is, those 8 s in which participants were asked “How much Time [or: Money] have you spent on your wristwatch?” and right before participants rated the product more positively in the time condition than in the money condition. BOLD responses during the time priming phase was directly compared to participants’ BOLD responses during the money priming phase. This approach of directly comparing time with money conditions is not only following the analyses of behavioral data by Mogilner and Aaker (2009) but is also in line with recent fMRI research, which directly compared different emotional states (e.g., Andersen et al., 2001) and different mindsets (e.g., Dietvorst et al., 2009) with each other.

First, we analyzed data on the single-subject level. Specifically, fixed-effects whole-brain general linear model (GLM) analyses were performed, using a regression model consisting of 14 predictors. A set of seven predictors corresponded to the specific phases of the task (i.e., an introduction phase, the first priming phase, and the five trial phases), while a set of seven confounding predictors captured motion-related artifacts and artificial activity within the ventricles (Weissenbacher et al., 2009). The BOLD signal change for each predictor was modeled by using a two-gamma hemodynamic response function (Friston et al., 1998).

Second, after creating statistical parametric maps for each participant by applying linear contrasts to the predictor estimates (i.e., betaweights), a random-effects GLM analysis was performed at the group level. At the group level, we employed a summary statistics approach, which uses the statistical maps computed at the single-subject level. This method takes the variability of effects across subjects into account, thus permitting population-level inferences. One between-subject factor (i.e., prime) with two levels (i.e., time and money) was generated to compare differences in activation for the predictor of interest (i.e., the “priming phase”). The global threshold was set to *p* < 0.01, uncorrected. Threshold maps were submitted to a region-of-interest-based correction for multiple comparisons. The correction criterion is based on Monte Carlo simulations calculating the likelihood of obtaining different cluster sizes. After 1,000 iterations, the minimum cluster size threshold that yielded a cluster-level false-positive rate of 0.05 was applied to the statistical maps (in our case seven voxel). Combined with relaxed single-voxel thresholds, this procedure will ensure a global error probability of *p* < 0.05 (Forman et al., 1995; Goebel et al., 2006).

Third, we compared predictors by performing a random-effects GLM analysis at regions of interest (e.g., Reimann et al., 2011). Regions of interests were defined both functionally and anatomically (Lancaster et al., 2000), and included both the right insula (at Talairach coordinates of *x* = 44, *y* = −26, *z* = 15) and the left insula (at Talairach coordinates of *x* = −31, *y* = −23, *z* = 18).

## Results

### Behavioral results

Building on the results of Mogilner and Aaker (2009), we expected a higher favorability rating in the time condition. To test this hypothesis, we ran a one-tailed independent-samples *t*-test to analyze whether participants rated their wristwatches more favorable in the time condition than in the money condition (the two-tailed test revealed non-significant differences). As expected, favorability was significantly greater in the time condition (*M*_time_ = 3.68, SD = 0.87) than in the money condition (*M*_money_ = 3.18; SD = 1.07), *t*(42) = 1.70, *p* < 0.05. These results replicate the behavioral findings of Mogilner and Aaker (2009), who found that when consumers are primed with time, their favorability ratings for products increase. However, data did not reveal replication of the effect for three other products (i.e., mobile phone, MP3 player, and laptop computer); in particular, differences were non-significant at *p* > 0.1.

### Neuroimaging results

**Figure 2:**
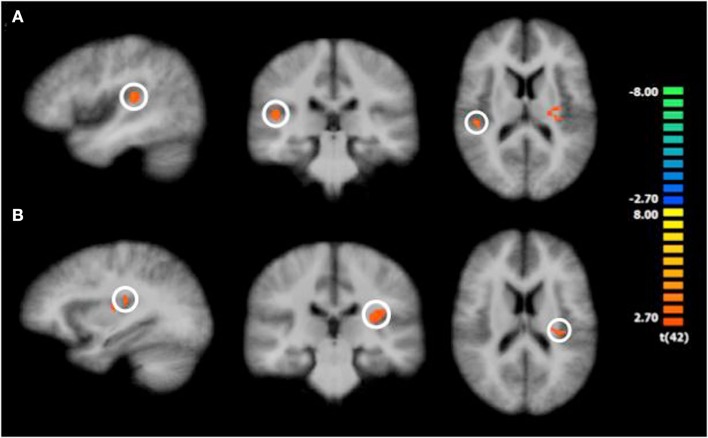
**Significantly increased activation in the insula following the time prime compared to the money prime**. Note: The color bar shows the *t*-values; colors from red to yellow indicate activation increases, and colors from blue to green indicate activation decreases. The insula is encircled. **(A)** shows right insula activation differences during the “priming phase,” and **(B)** shows left insula activation differences during the “priming phase.”

Consistent with prior research (e.g., Knutson et al., 2001a,b), we focused our analyses of the neuroimaging data on the trial phases in which emotional processes are most likely to operate: in our case, we concentrated on the actual priming phase. Contrasting BOLD responses during the time prime with BOLD responses during the money prime, whole-brain analysis results revealed increased activation in the right insula [*t*(42) = 3.39, *p *< 0.001], the left insula [*t*(42) = 4.18, *p* < 0.001], and the left medial temporal gyrus [*t*(42) = 3.88, *p* < 0.001]. The increases in insula and left medial temporal gyrus activation, therefore, preceded the time-versus-money effect. Figure 2 illustrates these activation changes, and Table 3 summarizes additional information, including Talairach coordinates and corresponding Brodmann areas. Further, we conducted a random-effects ROI analysis, focusing on activation changes in the insula. Results supported the findings from the whole-brain analysis, revealing greater activation in both the right insula [*t*(42) = 3.53; *p* < 0.05] and the left insula [*t*(42) = 4.13; *p* < 0.05] for time compared to money.

**Table 3:** **Activation changes for time compared to money during the “priming phase”**.

Brain area	Hemisphere	Brodmann area	*x*	*y*	*z*	*t*(42)	*p*
Insula	Right	13	44	−26	15	3.96	0.0003
Insula	Left	13	−31	−23	18	4.18	0.0001
Medial temporal gyrus	Left	21	−67	−44	0	3.88	0.0004

**N* = 43; random-effects general linear model. Brain areas, Brodmann areas, and Talairach coordinates (Talairach and Tournoux, 1988) refer to the peak activation voxel within each cluster of continuous voxels at a global threshold of *p *< 0.01 (uncorrected) and a minimum cluster threshold of seven voxel to ensure a total maximum false activation rate of 0.05*.

Purchasing a product implies paying a certain amount of money in exchange for it. Therefore, one might argue that with an increasing amount of money paid for a product, the amount of psychological pain associated with this product also increases when reminded (i.e., primed) of money (Prelec and Loewenstein, 1998; Soman, 2001). Because the insula also plays a role in processing negative emotions such as pain (Sawamoto et al., 2000), we had to account for such a possibility. Using the amount of money paid for the wristwatch as an approximation for the possible amount of pain felt, we analyzed the data using a linear regression model, which included the product rating as the dependent variable and the prime, actual amount paid, and time spent using the wristwatch as independent variables. We hypothesized that, if increasing amounts of money result in increased pain felt when money was made more salient, the product rating should be lower for higher amounts of money paid. We submitted data to a regression analysis to test if the amount of money paid predicted participants’ ratings of the wristwatch. The results of the regression indicated the predictor did not explain a significant proportion of variance in mean rating scores [*R*^2^ = 0.062, *F*(1,42) = 2.69, *p* > 0.10]. It was also found that the amount paid did not significantly predict mean rating scores, *b* = 0.24, *t*(42) = 1.64, *p* > 0.10. The result shows that the amount of money paid did not have a significant effect on the rating.

## Discussion

Prior research suggests certain mindset son which the time-versus-money effect is based. In particular, individuals in a temporal mindset apparently weigh emotional factors more heavily than individuals in a monetary mindset, who seem more objective in their processing (Mogilner and Aaker, 2009).

In this realm, the present research provides novel insight into the neurophysiological underpinnings of this effect. Our investigation shows that priming subjects with time (compared to money) is associated with significantly greater activation in the insula (both in the left and right hemisphere of the brain) and the left medial temporal gyrus.

The insula has been found to be a crucial brain region in diverse but related psychological phenomena, such as urging and addiction (Naqvi and Bechara, 2009), loss aversion (Knutson and Bossaerts, 2007; Knutson et al., 2007), interpersonal love (Bartels and Zeki, 2000, 2004; Beauregard et al., 2009), and brand love (Reimann et al., 2012). Conceptually, these psychological functions are closely related to Mogilner and Aaker’s (2009) notion of a time-versus-money effect, which argues in favor of a greater personal connection between consumer and product during time primes rather than during money primes. Besides the insula, other regions that might be expected to be associated with this task (i.e., other areas of the reward network or the amygdala, anterior cingulate cortex, and orbitofrontal cortex) did not show a significant neural difference between the time and money primes in our study. However, the medial temporal gyrus did show a substantial difference in activation between both primes. The medial temporal gyrus is part of a functional neuronal circuit that plays an important role in unconscious timekeeping and unconscious time estimation (Coull et al., 2004; Morillon et al., 2009). We speculate in saying that in a time prime greater usage of subject’s memory could have taken place. The time prime possibly led participants to more intense thoughts on the time spent with the product. Engaging participants more strongly in the stage of recapitulation of time may have resulted in unconscious time estimation and evaluation. Moreover, the medial temporal gyrus also appears to be related to craving; it has been found in connection with other brain areas in Goudriaan et al. (2010) and craving for basic needs like breathing (Liotti et al., 2001).

Thus, our result of increased activation in both the insula and the medial temporal gyrus in response to time primes may suggest that participants in the time condition were more attuned to an internal state of urging or product craving elicited by the prime than those in the money condition. Therefore, the finding from our neuroimaging experiment possibly explains the behavioral differences in product evaluations identified in previous research (Mogilner and Aaker, 2009) and replicated in this study. Because the time prime possibly elicited a more emotional mindset (as evidenced by increased insula activation – a limbic region) than the money prime, participants rated products as more favorable during the time prime than during the money prime. Because participants rated a product to which they had established a close relationship, the time prime could have increased such a feeling of closeness to the product, while the money prime could have decreased those feelings and possibly have triggered a feeling of distance. Indeed, previous behavioral work indicates that money mindsets lead to greater physical distancing (Vohs et al., 2006).

Besides making important contributions to research on the time-versus-money effect, the present study also has some limitations, which provide opportunities for future research. First, the insula has been found to be activated in cognitive tasks without a clear involvement of emotions, for example, working memory tasks (Cohen et al., 1997). Because the insula plays a role in many different emotional and motivational processes, the conceptual link between the identified neurophysiological processes and psychological phenomena, such as urging or relationship closeness, is not yet fully understood. Interesting questions could be answered from here: Are emotional mindsets induced by the time prime more similar to cravings or urges for additive substance than to other, less intense affective experiences? Would we observe similar activities in the insula when feelings of connection are directly induced without involving any priming of time?

Second, the medial temporal cortex has been found to be activated in many other functions unrelated to emotions, for example, the processing of written and heard language within the lexico-semantic network (De Zubicaray et al., 2001; Chen et al., 2002) and in conjunction with a broader neural network to episodic memory retrieval and encoding (Nyberg et al., 2000). This, in turn, could speak in favor of alternative explanations. As such, the role of the medial temporal gyrus in the time-versus-money effect needs further investigation. Future research may, for example, implement manipulations of urgings during time and money priming to further investigate the identified effects.

Third, we acknowledge that the term “mindset,” which was first used by Mogilner and Aaker (2009) in this context, may hold different meanings in different contexts. A mindset is often defined as an established set of assumptions, thoughts, and beliefs held by a group of people (Gollwitzer, 1990). In the case of time-versus-money primes, this definition is debatable, because of the relatively short temporal duration of mental states induced by priming (Isen and Gorgoglione, 1983; Bargh et al., 1988).

Fourth, we note that in this research the time-versus-money effect did not replicate behaviorally for several other products (i.e., mobile phone, MP3 player, and laptop computer), which is why we focused on one specific product for which it did replicate (here: the wristwatch). Future research could, thus, hone the experimental design to study the underlying processes of the time-versus-money effect for other product categories.

This research also provides implications for consumer behavior research. As Mogilner and Aaker (2009) already pointed out, research on time versus money highlights the power of contextual manipulations (e.g., option framing or choice set construction) to shift preferences (Simonson, 1989; Simonson and Tversky, 1992; Mogilner and Aaker, 2009). Because the psychological context in which attitudes are elicited seems to matter, it will be interesting for future research to further investigate the neural mechanism behind this kind of manipulation. Mogilner and Aaker (2009) further posited important implications for research on intrapersonal consistency. Specifically, they suggest that time might be a greater source of dissonance than money, and thus, a stronger driver of individuals’ ultimate attitudes. Our results support the point that time and money are not readily interchangeable in behavioral manipulations; both elicit different neural responses, suggesting a difference in the underlying mental processes operating when dealing with time compared to money. Boundary conditions of the time-versus-money effect imposed by different cultural backgrounds are another promising avenue to explore. Specifically, does the effect remain robust in cultures in which the meaning of money and time fundamentally differs?

From an applied perspective, this research provides novel insights for marketers on the underpinnings of the time-versus-money effect. The insight that an easily applied time prime could lead to an urge for a brand/product and, in turn, might increase brand/product likeability has important implications for the field of advertising.

Taken as a whole, this research speaks extensively to the diverse research community involved in research on time and money. Even though our research might raise a new set of questions, we believe that it may also provide meaningful answers on some of the psychological and neurophysiological differences between time and money.

## Conflict of Interest Statement

The authors declare that the research was conducted in the absence of any commercial or financial relationships that could be construed as a potential conflict of interest.
